# Usefulness of water pressure observation in detection and direct clipping of source of colonic diverticular bleeding

**DOI:** 10.1055/a-2081-6798

**Published:** 2023-05-26

**Authors:** Jun Takada, Masamichi Arao, Kentaro Kojima, Sachiyo Onishi, Masaya Kubota, Takashi Ibuka, Masahito Shimizu

**Affiliations:** Department of Gastroenterology, Gifu University Graduate School of Medicine, Gifu, Japan

Colonic diverticular bleeding is the most common cause of bleeding in the lower gastrointestinal tract 1 . Endoscopic identification of stigmata of recent hemorrhage (SRH), such as active bleeding, adherent clot, or visible vessel, is important but often difficult 2 3 . The waterjet scope, the use of which has been reported to be an independent predictive factor for identifying SRH, is primarily used to wash away feces or clots inside the diverticulum 4 . Herein, we describe a useful case of water pressure observation for the detection of SRH and endoscopic direct clipping of diverticular bleeding.

A 75-year-old man with hematochezia had undergone repeated colonoscopies. Multiple diverticula were observed in the right colon, but spontaneous hemostasis prevented the identification of bleeding points. Therefore, the patient was transferred to our hospital. He developed hematochezia again 2 days later. We performed an urgent colonoscopy with bowel preparation.

We used a colonoscope with waterjet function (PCF-H290TI; Olympus Medical Systems, Tokyo, Japan). We attached a super-soft transparent hood without slits (Space adjuster; TOP, Tokyo, Japan). Fresh blood and multiple diverticula with hemostatic clips applied by a previous physician were observed in the right colon; however, the bleeding stopped spontaneously ( Fig. 1 a ). We placed the tip hood of the scope in close contact with each diverticulum and inflated it with water. The water pressure opened the diverticulum, which facilitated observation of the interior. We detected a pulsating visible vessel within a diverticulum in the ascending colon ( Fig. 1 b ). Although the diverticulum could not be inverted, we inflated it with water to create a working space that allowed direct pinpoint clipping with a short-armed hemostatic clip (HX-610-135XS; Olympus) ( Fig. 2 , Video 1 ).

**Fig. 1:**
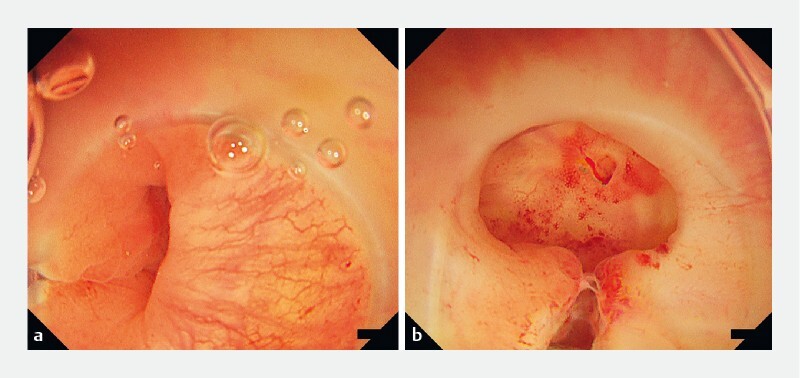
Endoscopic view of the bleeding source inside the diverticulum. **a** Before water pressure observation. **b** After water pressure observation; a pulsating visible vessel was detected.

**Fig. 2:**
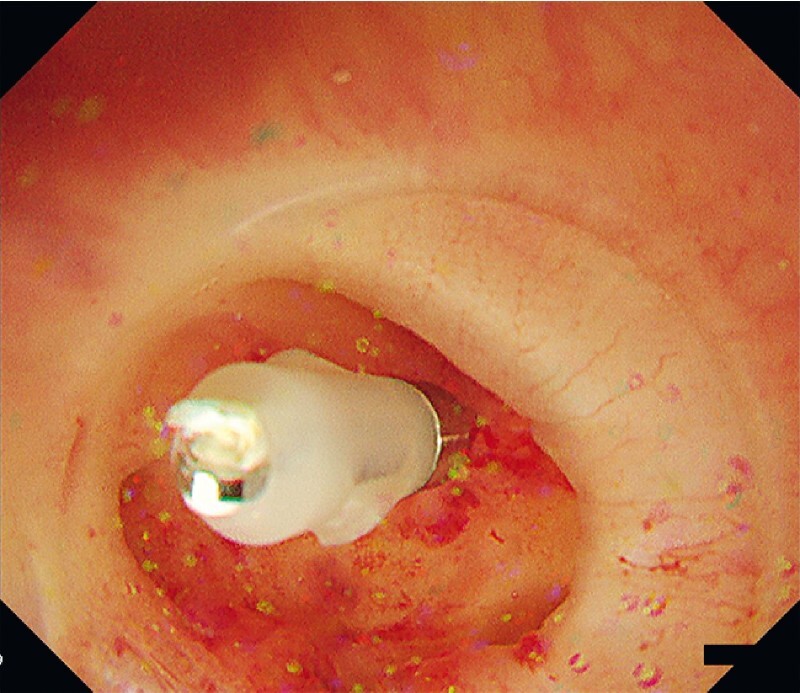
Successful direct pinpoint clipping with a short-armed hemostatic clip.

**Video 1** Water pressure observation for detection and direct clipping of source of colonic diverticular bleeding.


The patient was discharged without additional intervention. The patient had no rebleeding during the 2-month follow-up period.

Endoscopy_UCTN_Code_TTT_1AQ_2AZ

## Notes

Endoscopy E-Videos

https://eref.thieme.de/e-videos
*E-Videos* is an open access online section of the journal *Endoscopy* , reporting on interesting cases and new techniques in gastroenterological endoscopy. All papers include a high-quality video and are published with a Creative Commons CC-BY license. Endoscopy E-Videos qualify for HINARI discounts and waivers and eligibility is automatically checked during the submission process. We grant 100% waivers to articles whose corresponding authors are based in Group A countries and 50% waivers to those who are based in Group B countries as classified by Research4Life (see: https://www.research4life.org/access/eligibility/ ). This section has its own submission website at https://mc.manuscriptcentral.com/e-videos
